# Effect of a hospital command centre on patient safety: an interrupted time series study

**DOI:** 10.1136/bmjhci-2022-100653

**Published:** 2023-01-25

**Authors:** Teumzghi F Mebrahtu, Ciarán D McInerney, Jonathan Benn, Carolyn McCrorie, Josh Granger, Tom Lawton, Naeem Sheikh, Rebecca Randell, Ibrahim Habli, Owen Ashby Johnson

**Affiliations:** 1 School of Computing, University of Leeds, Leeds, UK; 2 Bradford Institute for Health Research, Bradford, UK; 3 Wolfson Centre for Applied Health Research, Bradford Royal Infirmary, Yorkshire and Humber Patient Safety Translational Research Centre, Bradford, UK; 4 School of Psychology, University of Leeds, Leeds, West Yorkshire, UK; 5 Bradford Royal Infirmary, Bradford Teaching Hospitals NHS Foundation Trust, Bradford, UK; 6 Faculty of Health Studies, University of Bradford, Bradford, UK; 7 Bradford Royal Infirmary, Wolfson Centre for Applied Health Research, Bradford, UK; 8 Department of Computer Science, University of York, York, UK

**Keywords:** health services research, information technology, health information systems

## Abstract

**Background:**

Command centres have been piloted in some hospitals across the developed world in the last few years. Their impact on patient safety, however, has not been systematically studied. Hence, we aimed to investigate this.

**Methods:**

This is a retrospective population-based cohort study. Participants were patients who visited Bradford Royal Infirmary Hospital and Calderdale & Huddersfield hospitals between 1 January 2018 and 31 August 2021. A five-phase, interrupted time series, linear regression analysis was used.

**Results:**

After introduction of a Command Centre, while mortality and readmissions marginally improved, there was no statistically significant impact on postoperative sepsis. In the intervention hospital, when compared with the preintervention period, mortality decreased by 1.4% (95% CI 0.8% to 1.9%), 1.5% (95% CI 0.9% to 2.1%), 1.3% (95% CI 0.7% to 1.8%) and 2.5% (95% CI 1.7% to 3.4%) during successive phases of the command centre programme, including roll-in and activation of the technology and preparatory quality improvement work. However, in the control site, compared with the baseline, the weekly mortality also decreased by 2.0% (95% CI 0.9 to 3.1), 2.3% (95% CI 1.1 to 3.5), 1.3% (95% CI 0.2 to 2.4), 3.1% (95% CI 1.4 to 4.8) for the respective intervention phases. No impact on any of the indicators was observed when only the software technology part of the Command Centre was considered.

**Conclusion:**

Implementation of a hospital Command Centre may have a marginal positive impact on patient safety when implemented as part of a broader hospital-wide improvement programme including colocation of operations and clinical leads in a central location. However, improvement in patient safety indicators was also observed for a comparable period in the control site. Further evaluative research into the impact of hospital command centres on a broader range of patient safety and other outcomes is warranted.

**WHAT IS ALREADY KNOWN ON THIS TOPIC**

- Although command centres have been introduced in hospitals in developed world countries, their impact on patient safety remains unknown.

**WHAT THIS STUDY ADDS**

- A command centre that includes introduction of both technological (data display) elements and organisational components may improve patient safety. However, it appears that the majority of the impact may result from the processes around the command centre itself rather than the technological aspect.

**HOW THIS STUDY MIGHT AFFECT RESEARCH, PRACTICE OR POLICY**

- It is a common belief that command centres can improve patient safety. However, this has not been supported by patient safety metrics examined for this study. Hence, further research is warranted.

## Introduction

Fragmented healthcare is neither cost-effective nor safe for the delivery of patient care.^1 2^ In most UK National Health Service (NHS) hospitals, health service delivery is fragmented across multiple departments and services with major implications for patient safety, efficiency and good patient care. Such fragmentation can, however, be minimised using health information technology to improve the flow of information—between and within healthcare providers.^3 4^ The idea of improving communication by using digital information systems to centralise information to improv*e* situational awareness was pioneered by the National Aeronautics and Space Administration (NASA) for the purpose managing space flights six decades ago.^5^ This central system, also known as ‘command centre’ or ‘mission control’ has been widely adopted in retail industries, finance and banking, automotive, manufacturing and transport industries and to a lesser degree within the healthcare sector.

In the last 5 years, a number of hospitals in Canada, China, the UK, USA and Saudi Arabia have been piloting ‘command centres’ for the purpose of patient-flow management. Although not from systematically conducted studies, preliminary reports suggest that command centres have a positive impact on patient care delivery process.^6–10^ For example, in Johns Hopkins Hospital USA, patient transfers from other hospitals improved by 46%, ambulances dispatches reduced by 43 min and bed allocation for emergency admission patients reduced by 3.5 hours.^7^

In the UK, there are currently only four NHS hospital trusts who are piloting command centres. One of these is Bradford Teaching Hospitals NHS Foundation Trust, which provides hospital services for around half a million people. In 2019, the Trust introduced a command centre at its main hospital, Bradford Royal Infirmary (BRI).^11^ The command centre is made up of software and display screens (also known as ‘tiles’) that provides real-time information (updated every 3 min) and alerts for patient care and intervention across the hospital site, including: overall hospital capacity, emergency department status, patient transfers, discharge tasks, care progression and patient deterioration. Information is inputted by the staff in the departments of the BRI hospital as part of normal care processes within the electronic patient record system and is automatically reconfigured to be shown in defined parameters within each of the tiles.

The Bradford Command Centre aims to provide safer care by addressing increasing pressure in the ED and associated challenges downstream related to capacity and demand, monitoring patients for placement in most appropriate care settings and access to real-time information required to make decisions. Such command centres have the potential to improve future patient flow and safety, and research to understand the health service delivery, safety and operational factors is considered an area of major importance for hospitals. We hypothesised that the implementation of an integrated and centralised hospital command centre improves patient safety. Therefore, our study aim was twofold: (1) to investigate the impact of Bradford command centre on patient safety outcomes in BRI hospital (2) to compare the pattern of patient safety outcomes of BRI hospital with Calderdale & Huddersfield Hospitals (CHH) which is without a command centre.

## Methods

### Study population

Participants of the study were patients who visited accident and emergency and unplanned admissions at the intervention site, BRI hospital Trust, where the command centre was introduced and a nearby and similar sised hospital, CHH, which we used as a control. The study period was between 1 January 2018 and 31 August 2021 and covered the period before, during and after the implementation of the command centre at the intervention site.

### Study design

This is a retrospective population-based cohort study undertaken as part of a mixed method evaluation project with a formal evaluation protocol published by the authors in January 2022.^12^ Qualitative study of the command centre programme gave rise to two hypothesised intervention timelines, one focusing on the implementation and activation of the technological components of the command centre and the other ‘complex’ intervention model that sought to account for the broader patient flow and operational redesign programme in which the command centre technology was a part. For the technology model, a three-phase, interrupted time series model was used to reflect incremental implementation of the visual displays in the command centre, consisting of a preintervention (baseline), first intervention component (‘command centre displays roll-in’) and second intervention component (‘command centre activation’). For the complex intervention model, a five-phase, interrupted time series model was used that consisted of preintervention (baseline), first intervention component (‘onset of patient flow programme’), second intervention component (‘command centre displays roll-in’), third intervention component (‘command centre activation’) and fourth intervention component (‘hospital wide engagement and training’), the latter referring to roll-out of remote access to command centre data across the hospital. See table 1 for the details of the timeline and interrupts.

**Table 1:** Project timeline and intervention phases

Date	Event
1 January 2018	Start of study
1 July 2018	Onset of patient flow programme
1 May 2019	Command centre displays roll-in
1 December 2019	Command centre activation and hospital wide engagement and training commences
1 May 2021	Post-COVID-19 resumption of hospital wide engagement and training
31 August 2021	End of study

### Data source

UK NHS Digital Secondary Use Services (SUS) data were used. These data are secure, patient-level data that is sent by both hospitals to NHS England to support national tariff policy and secondary analysis. Construction of the SUS data was conducted by Connected Bradford, a team located at the Bradford Institute for Health Research (https://www.bradfordresearch.nhs.uk/our-research-teams/connected-bradford/). The team uploaded the SUS data onto a Google Cloud Platform where relevant data were processed before final outputs were extracted.^13^

### Patient and public involvement and engagement

Public and patient representatives contributed to the development of the research protocol and towards selection of proxy patient safety outcomes. Details of the wider project’s patient and public involvement and engagement are available in the published protocol.^12^

### Outcome variables

Potential patient safety outcome indicators were listed in our published protocol.^12^ However, due to data availability, three indicators for the BRI hospital (mortality, readmissions within 72 hours and postoperative sepsis) and two indicators for the CHH (mortality and readmissions within 72 hours) were analysed.

The proportions of readmissions and mortality in hospital were calculated as the total readmissions and death among emergency admissions, respectively, divided by the total number of emergency admissions. Postoperative sepsis was calculated by dividing the weekly count of patients with sepsis diagnostic codes in their records by the count of surgical operations conducted in that week. The list of surgical operation codes was extracted from the UK Health Security Agency published document.^14^ Postoperative sepsis occurrences were identified using T814 ICD10 code.^15^

### Variables for analysis

Dummy variables were created for each of the intervention components (‘Onset of patient flow programme’, ‘command centre displays roll-in’, ‘command centre activation’ and ‘hospital wide engagement and training’), COVID-19 pandemic and spikes of COVID-19 pandemic.^16^ The components of the intervention were given a value of ‘1’ starting from the date of its introduction until the introduction of the next component or phase, then a value of ‘0’ for the rest of the period. ‘COVID-19 pandemic’ was given a value of ‘0’ through February 2020 and a value of ‘1’, thereafter. A spike dummy variable was also added by setting ‘1’ for the COVID-19 spike periods based on the UK data^16^ and ‘0’ throughout.

A continuous incremental time variable was coded from the start of the time series (eg, 1, 2, 3, 4). The intervention phases were also modelled using five continuous time variables with ‘0’ in the preintervention period, ‘1, 2, 3, 4….’ from the onset of the intervention phase. In addition, seasonality was modelled by including dummy variables for the number of weeks in the year.

### Statistical analysis and software

First, outcome variables were summarised descriptively. Then, to assess the impact of the command centre on patient safety outcomes, linear regression interrupted time series analysis was used.^17^ Linear time series models were fitted to the BRI and CHH data separately. Tests for serial autocorrelation of residuals were conducted and all tests were non-statistically significant. Hence, regression models with autoregressive integrated moving average (ARIMA) errors were not used. To compare the changes (ie, beta coefficients) of two outcomes (mortality and readmissions within 72 hours) in BRI and CHH, the difference of the changes (beta coefficient BRI subtracted beta coefficient CHH) and total variances (variance BRI+variance CHH) were calculated to derive the difference in changes and their CIs.^18^

Akaike information criterion^19^ and Bayesian information criterion^20^ were used to select the best fitting models. Analyses were implemented in R (V.4.0.2). We adopted 5% significance levels and 95% CIs throughout.

A five-phase interrupted time series was used for the main analyses. To explore if the command centre activation alone would have had an impact on outcomes, a three-phase interrupted time series model was used for sensitivity analyses for the intervention site (BRI hospital), including the display roll-in period and activation event.

## Results

### Descriptive summary

**Figure 1:**
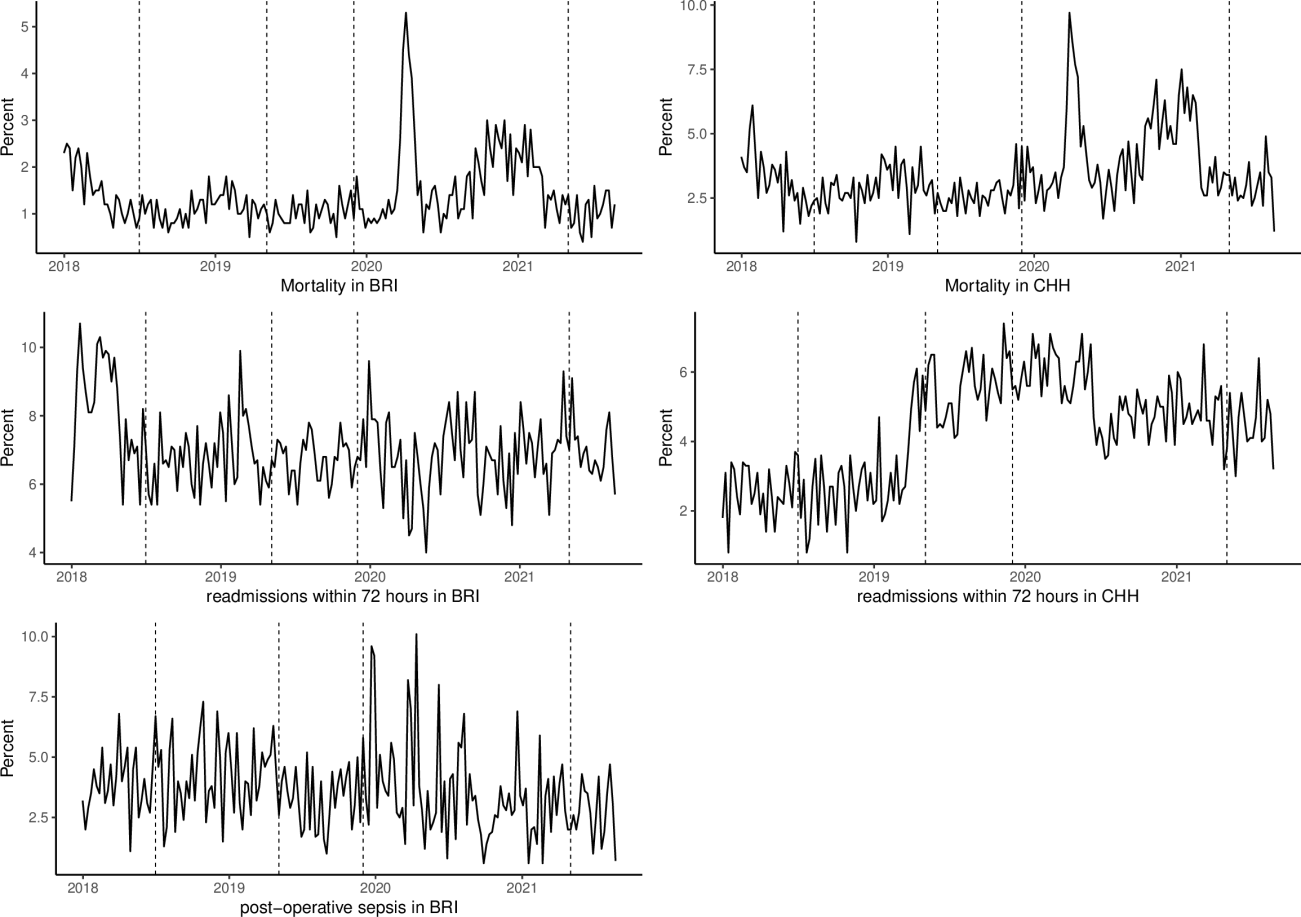
An overall pattern of patient safety indicators during the study period. BRI, Bradford Royal Infirmary; CHH, Calderdale & Huddersfield Hospitals.

There were a total of 203 807 in-patient emergency admissions and 34 625 operations performed in BRI hospital and 291 018 in-patient emergency admissions in CHH during the study period. The weekly mortality (as a percentage of weekly admissions) stayed below 3% and 5% (in BRI and CHH, respectively) for most of the study period except a sudden increase in March–April 2020 when a spike in the hospital admissions associated with COVID-19 was reported in the UK.^16^ The weekly deaths appear to be higher for the period after COVID-19 pandemic when compared with the prepandemic period. Overall, the average mortality throughout the study period was higher in CHH than BRI (see figure 1 and table 2).

**Table 2:** Summary of patient safety indicators, mean (SD)

Period	Mortality (%)*		Readmissions within 72 hours (%)*		Post-operative sepsis (%)†
	BRI	CHH	BRI	CHH	BRI
1 January 2018–30 June 2018 (pre-intervention)	1.5 (0.56)	3.3 (1.1)	8.2 (1.5)	2.5 (0.7)	3.8 (1.2)
1 July 2018–30 April 2019 (patient flow programme)	1.1 (0.31)	2.9 (0.78)	6.8 (0.97)	2.9 (1.22)	4.2 (1.6)
1 May 2019–30 November 2019 (command centre display roll-in)	1.0 (0.29)	2.5 (0.57)	6.7 (0.62)	5.6 (0.87)	3.2 (1.2)
1 December 2019–30 April 2021 (command centre activation)	1.7 (0.94)	4.3 (1.67)	6.8 (1.1)	5.2 (0.93)	3.6 (2.1)
1 May 2021–31 August 2021 (engagement resumption)	1.1 (0.38)	2.9 (0.83)	6.9 (0.81)	4.5 (0.84)	2.7 (1.2)

*Values are percentages with respect to weekly counts of in-patient emergency admissions.

†Values are percentages with respect to weekly counts of surgical operations.

BRI, Bradford Royal Infirmary hospital; CHH, Calderdale & Huddersfield Hospitals.

The weekly readmissions within 72 hours (as a percentage of the total emergency admissions) remained above 6% and 4% (in BRI and CHH, respectively) for the majority of the study period. The average readmissions in BRI hospital were just over 8% during the first 6 months of the study period and stayed just under 7% for the remaining period. On the other hand, the readmissions in the CHH were under 3% during the first 16 months, then nearly doubled during the rest of the study period. The patterns of the weekly readmissions do not appear to have been greatly affected by the pandemic. Overall, the weekly readmissions were higher in BRI than CHH (see figure 1 and table 2).

The weekly postoperative sepsis (as a percentage all surgical operations performed) stayed between 1.5% and 6% for majority of the period with occurrence of spikes during January and April 2020 (see figure 1). The overall postoperative sepsis ranged between 0.6% and 10%, and it was below 5% during the study period on average (see figure 1 and table 2).

### The effect of intervention

#### Main analyses (five-phase interrupted time series)

**Figure 2:**
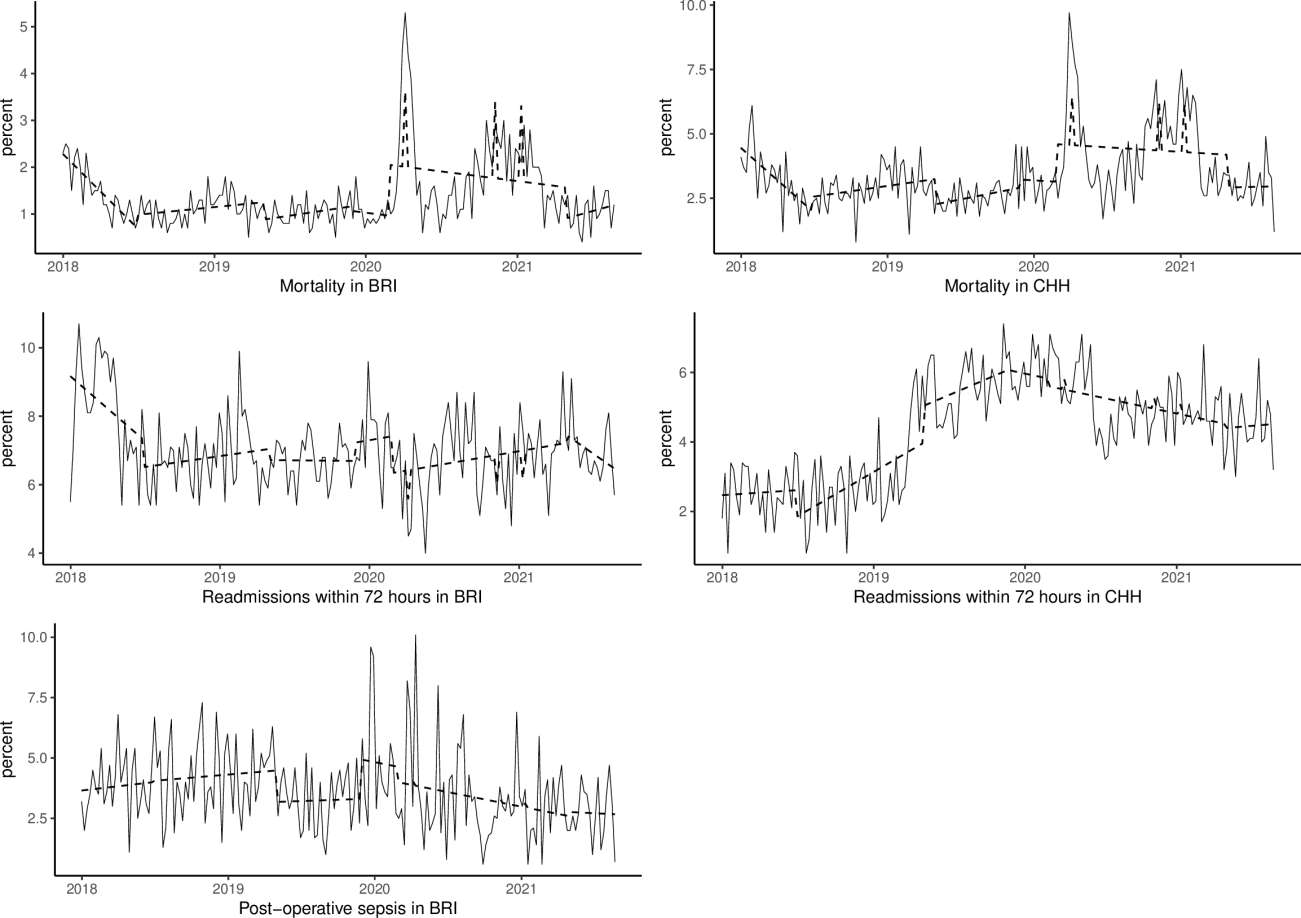
Actual values (solid lines) and model estimated values (dashed lines) of outcome indicators. BRI, Bradford Royal Infirmary; CHH, Calderdale & Huddersfield Hospitals.

In BRI hospital, when compared with the preintervention period, the weekly mortality decreased by 1.4% (95% CI 0.8% to 1.9%), 1.5% (95% CI 0.9% to 2.1%), 1.3% (95% CI 0.8% to 1.9%) and 2.5% (95% CI 1.7% to 3.4%) at onset of the first (‘patient flow programme’), second (‘command centre display roll-in’), third (‘command centre activation’) and fourth (‘hospital-wide engagement resumption’) intervention periods, respectively. At onset of the first, second and third intervention periods, the weekly per cent of readmission within 72 hours also decreased by 2.7% (95% CI 1.7% to 3.8%), 2.5% (95% CI 1.4% to 3.6%), 2.0% (95% CI 1.0% to 3.0%) and 0.7% (95% CI 2.2% to 0.9%), respectively. The weekly postoperative sepsis did not show a significant change during the study period, however (see table 3 and figure 2).

**Table 3:** Summary results for five-phase models

Outcome	Intervention phase	Change in BRI (95% CI)*	Change in CHH (95% CI)*	Difference between sites (BRI−CHH), 95% CI †
Mortality (%)	Pre-intervention	Ref.	Ref.	
	Patient flow programme	−1.4 (−1.9 to −0.8)	−2.0 (−3.1 to −0.9)	0.6 (−0.6 to 1.9)
	Command centre display roll-in	−1.5 (−2.1 to −0.9)	−2.3 (−3.5 to −1.1)	0.8 (−0.6 to 2.2)
	Command centre activation	−1.3 (−1.82 to −0.7)	−1.3 (−2.4 to −0.2)	0.04 (−1.2 to 1.3)
	Engagement resumption	−2.5 (−3.4 to −1.7)	−3.1 (−4.8 to −1.4)	0.6 (−1.4 to 2.5)
Readmissions within 72 hours (%)	Pre-intervention	Ref.	Ref.	
	Patient flow programme	−2.7 (−3.8 to −1.7)	−0.6 (−1.5 to 0.2)	−2.1 (−3.4 to −0.7)
	Command centre display roll-in	−2.5 (−3.6 to −1.4)	2.6 (1.6 to 3.5)	−5.1 (−6.6 to −3.6)
	Command centre activation	−2.02 (−3.0 to −1.0)	3.6 (2.7 to 4.5)	−5.6 (−6.9 to −4.3)
	Engagement resumption	−0.70 (−2.3 to 0.9)	2.2 (0.8 to 3.5)	−2.9 (−4.8 to −0.8)
Post-operative sepsis (%)	Pre-intervention	Ref.	–	–
	Patient flow programme	0.4 (−1.2 to 2.0)	–	–
	Command centre display roll-in	−0.5 (−2.2 to 1.3)	–	–
	Command centre activation	1.31 (−0.3 to 2.9)	–	–
	Engagement resumption	−0.2 (−2.7 to 2.2)	–	–

*Models were adjusted for trend, COVID-19 pandemic (pre-pandemic and post-pandemic) and COVID-19 spikes.

†Calculated as difference in changes (change BRI−change CHH) and total variances (variance BRI+variance CHH).

BRI, Bradford Royal Infirmary hospital; CHH, Calderdale & Huddersfield Hospitals.

In CHH, compared with the baseline, the weekly mortality decreased by 2.0% (95% CI 3.1 to 0.9), 2.3% (95% CI 3.5 to 1.1), 1.3% (95% CI 2.4 to 0.2), 3.1% (95% CI 4.8 to 1.4) for the respective intervention phases of BRI hospital. However, except for the first intervention period, readmissions within 72 hours showed a significant increase during the second (change=2.6%, 95% CI 1.6 to 3.5), third (change=3.6, 95% CI 2.7 to 4.5) and forth (change=2.2, 95% CI 0.8 to 3.5), see table 3 and online supplemental table 1.

10.1136/bmjhci-2022-100653.supp1 Supplementary data

When the BRI hospital and CHH are compared in terms of indicator outcome changes during the study period, the weekly mortality significantly improved while the weekly readmissions showed improvement in BRI hospital but not in CHH (see table 3).

#### Sensitivity analysis (three-phase interrupted time series)

When implementation and activation of the technological aspects of the command centre were modelled, there was no significant difference between the pre and postintervention periods in the patient safety indicators. For example, mortality did not significantly change after the ‘command centre display roll-in’ (change=−0.5%, 95% CI −1.3 to 0.3) and ‘command centre activation’ (change=−0.3, 95% CI −1.0 to 0.4) periods when compared with the preintervention period (see online supplemental table 2).

## Discussion

In this preintervention and postintervention comparative study using SUS data, the findings indicate that introduction of the Bradford Command Centre may have improved patient safety. However, given improvements in mortality have also been observed in the CHH (control site) during the same period, improvements seen in the BRI hospital data may not be entirely due to the command centre. In addition, there was no significant difference between preintervention and postintervention periods linked to only the technological components of the command centre system.

Hospital command centres are expected to improve the management of patient flow by making use of real-time monitoring of patients. It is hypothesised that this improved patient flow is beneficial for patient safety. However, in our related work,^21^ we found that measures of patient flow did not indicate improvements at the BRI site during the study period. This suggests that patient flow may not be primarily responsible for the improvements. Given also that similar improvements were seen at our control site, it could be that the changes observed by our measures of patient safety were due to nationwide responses to the COVID-19 pandemic or some other within-hospital factors that we did not measure. The impact of command centres on patient safety in complex multiple department hospitals is rarely reported in the literature, mainly due to the novelty of this type of initiative in acute care. A recent report from Saudi National Health Command Centre (NHCC) indicated that emergency admissions mortality was below 2%.^6^ The mortality rate reported by the authors agrees with our findings of this study. What must be noted though is that the NHCC is a hub at a national level and its report appears to have compared pre-COVID-19 and post-COVID-19 pandemic data. On the other hand, the Bradford command centre is a single trust hospital and our study has compared preintervention period data against the multistage postintervention data, which included the prepandemic and postpandemic period.

Our study has certain limitations. First, health service delivery was significantly affected by the COVID-19 pandemic resulting in rapid systemwide effects, which may have impacted on the population of patients and capacity management in both hospitals. Cancellation and postponement of surgical operations were common due to reallocation of resources during the peaks of the pandemic. Although we attempted to control for the effects of the pandemic in our time series models, the proximity of the activation of the command centre with the onset of the pandemic surge makes it difficult to isolate the effect of the intervention or control for the pandemic without masking potential variation.

Second, apart from the command centre, it has been assumed that the intervention site (BRI hospital) and control site (CHH) are equivalent in other factors, which may not necessarily be the case. The control site showed considerably higher initial mortality which might have led to subsequent reduction in mortality rates or local interventions to reduce mortality, acting as a confounding factor in attempts to isolate the effect of the command centre intervention. Readmission rates additionally showed widely different trends between the study and control site.

Another potential limitation of the study concerns the focus of this quantitative evaluation on a small number of outcome indicators for what was a system-wide initiative designed to impact many areas. Although informing our intervention models using qualitative research at the study site is a strength in our design, qualitative investigation additionally revealed the complexity of this type of intervention and the challenges of implementation within a pressured acute care environment. This may have influenced the study outcome in a number of ways. Staff recall of the historical implementation timeline was variable (especially for piloting and roll-in of intervention components, including organisational in addition to technological elements). There were suggestions that colocation of staff in the command centre room preceded the roll-in and activation phase for command centre displays, so the team may have already been established and coordinating functions sooner than the intervention timeline suggests, leading to under specification of our model. When considering the challenges observed in implementing the technological aspects of the intervention, including data quality, there may have been significant time lag between activation of components and any impact on patient safety outcomes. Given the complexity in our intervention model, we did not seek to control for lagged effects of intervention implementation (the time it takes for an intervention to start to influence detectable outcomes). Rather, we presumed that the effects of the intervention components were instantaneous.

Finally, due to data access limitations, we were not able to explore all outcomes identified for analysis in our study protocol. Hence, evaluation is needed, across multiple healthcare systems and command centre models, to understand how this type of intervention impacts downstream patient safety outcomes.

Nonetheless, the strengths of the study are threefold. First, we have used a large sample size for the analyses: a total of inpatient 203 807 inpatient visits and 34 625 surgical operations. Second, the use of electronic health record data minimises the inherent biases and errors in other types of observational data. Third, we employed a robust quasi-experimental design using repeated time series measurement.

In conclusion, the results of the study indicate that a digital hospital command centre package that includes both technological (data display) elements and organisational components may have a marginal positive impact on some patient safety outcomes. However, patient safety improvements in the control site hospital suggest that it may not entirely be due to the introduction of the command centre. In addition, when the technology alone was considered as the intervention (command centre display roll-in and command centre activation), it does not appear to have a significant impact on patient safety outcomes. Thus, further research using data from other hospital organisations that use command centres is warranted.

## Data Availability

All data relevant to the study are included in the article or uploaded as supplementary information.
