# Supplementary material for: Effect of a hospital command centre on patient safety: an interrupted time series study
Source: BMJ Health Care Inform. 2023 Jan 25;30(1):e100653. doi: 10.1136/bmjhci-2022-100653 (PMC9884873; doi:10.1136/bmjhci-2022-100653)
Supplement: Supplementary data [file bmjhci-2022-100653supp001.pdf]

Supplementary Table 1: summary results for five phase models

Outcome	Trend (trend and SE) in BRI	Trend (trend and SE) in CHH
Mortality (%) †	Patient flow program= 0.01(0.01)	Patient flow program= 0.02(0.01)
	Command centre display roll-in = 0.01(0.01)	Command centre display roll-in = 0.02(0.02)
	Command centre activation= **-0.01(0.00)**	Command centre activation= **-0.01(0.01)**
	Engagement resumption**=** 0.01(0.03)	Engagement resumption**=** 0.00(0.06)
Readmissions within 72 hours (%)†	Patient flow program= 0.01(0.01)	Patient flow program= 0.05(0.02)
	Command centre display roll-in = -0.00(0.02)	Command centre display roll-in = 0.04(0.02)
	Command centre activation= 0.01(0.01)	Command centre activation= -0.02(0.01)
	Engagement resumption= -0.06(0.05)	Engagement resumption= 0.01(0.04)
Post-operative sepsis (%)	Patient flow program= 0.01(0.02)	-
	Command centre display roll-in = 0.00(0.03)	-
	Command centre activation= -0.02(0.01)	-
	Engagement resumption**= 1.17(0.52)**	-

Note: *, reference is the pre-intervention period; [a]; models were adjusted for baseline trend, covid-19 pandemic (pre- and post-pandemic) and covid-19 spikes †, inpatient emergency admissions; ‡, accident and emergency visits.

Supplementary Table 2: Summary results for three-phase models

Outcome	Intervention phase	Effect change (95% CI)
Mortality (%)†	Pre-intervention	Ref.
	Command centre tile roll-in	-0.50(-1.3 to 0.27)
	Command centre activation	-0.32(-1.0 to 0.39)
Readmissions within 72 hours (%)†	Pre-intervention	Ref.
	Command centre display roll-in	-0.17(-1.5 to 1.2)
	Command centre activation	0.36(-0.9 to 1.6)
Post-operative sepsis (%)	Pre-intervention	Ref.
	Command centre display roll-in	-0.58(-2.7 to 1.5)
	Command centre activation	1.17(-0.8 to 3.1)

Note: *, models were adjusted for trend, COVID-19 pandemic (pre- and post-pandemic) and COVID-19 spikes; †, inpatient emergency admissions of BRI hospital; ‡, accident and emergency visits of BRI hospital.
